# Twist and shout: magnetic resonance imaging findings in ovarian torsion

**DOI:** 10.1590/0100-3984.2018.0079

**Published:** 2019

**Authors:** Matheus Menezes Gomes, Larissa Sobral Cavalcanti, Rainier Luz Reis, Eduardo Just da Costa e Silva, Joanna Braynner Dutra, Andréa Farias de Melo-Leite

**Affiliations:** 1 Instituto de Medicina Integral Professor Fernando Figueira, Recife, PE, Brazil.; 2 Hospital das Clínicas de Pernambuco da Universidade Federal de Pernambuco (UFPE), Recife, PE, Brazil.; 3 Hospital da Mulher do Recife, Recife, PE, Brazil.; 4 Clínica Lucilo Ávila Jr., Maximagem, Recife, PE, Brazil.

**Keywords:** Magnetic resonance imaging, Ovary, Torsion abnormality, Ovarian neoplasms, Ovarian cysts

## Abstract

Adnexal torsion is characterized by partial or complete rotation of the suspensory ligament of the ovary and its corresponding vascular pedicle, resulting in vascular impairment that can culminate in hemorrhagic infarction, as well as necrosis of the ovary and fallopian tube. Because there are myriad causes of acute pelvic pain, the differential diagnosis of ovarian torsion is often challenging. Consequently, radiologists should be familiar with the main imaging findings. In this regard, there are typical signs of ovarian torsion on magnetic resonance imaging, including increased ovarian volume with stromal edema and peripheral distribution of the ovarian follicles, as well as thickening of the fallopian tube, an adnexal mass (causal factor) that shifts toward the midline, and the classic, pathognomonic “whirlpool sign”. The objective of this essay was to review and illustrate the various magnetic resonance imaging findings in ovarian torsion.

## INTRODUCTION

Adnexal torsion is characterized by partial or complete rotation of the suspensory ligament of the ovary and its corresponding vascular pedicle, resulting in rotation of the ovary, fallopian tube, or both, which impairs blood flow and can culminate in necrosis of those structures^(1)^. Although it is considered the fifth most common gynecological emergency in adult women^(2)^, its diagnosis is often made difficult by a clinical presentation that is considered nonspecific. Concomitant causes of acute pelvic pain can also mask the symptoms of ovarian torsion.

Ultrasound is a well-established screening method for gynecological emergencies. However, because of the technical limitations and low tissue resolution of ultrasound, complementary examinations, primarily magnetic resonance imaging (MRI), are often required for the diagnosis of adnexal torsion. Therefore, it is of fundamental importance that radiologists are familiar with the different presentation forms of this gynecological condition.

The objective of this essay is to illustrate and review the various MRI findings in ovarian torsion, based on images obtained from the case files archived at our facility.

## ANATOMY AND PATHOPHYSIOLOGY

The ovary is connected to the pelvic wall by the infundibulopelvic ligament (suspensory ligament of the ovary), which holds it lateral or superior to the uterus. The ovarian blood vessels run along the suspensory ligament. The medial aspect of the ovary is connected to the uterus by the ovarian ligament, which is composed of muscle and fibrous tissue, vascularization being provided by the uterine artery^(3)^.

When an ovarian cyst or mass causes rotation of the suspensory and uterine ligaments, the resulting torsion usually affects the ipsilateral ovary and fallopian tube^(3)^. Although torsion of the suspensory ligament immediately reduces the venous and lymphatic flows of the corresponding ovary, the arterial influx is initially preserved because the arteries have thick, muscular walls and are less likely to collapse. Consequently, the affected ovary can show edema and an increase in volume, which, over time (after a progressive increase in pressure), are followed, in succession, by arterial thrombosis, ischemia, and infarction. In cases of partial torsion, the capillary hydrostatic pressure remains elevated and obstructs lymphatic drainage, leading to massive ovarian edema^(4-7)^.

## CLINICAL PROFILE

The symptoms of ovarian torsion are often nonspecific, making it difficult to differentiate it from other causes of acute abdominal pain. The classic presentation includes unilateral pelvic pain, a palpable mass, and signs of peritoneal irritation. Nonspecific symptoms such as nausea and vomiting can also be present. In some cases, patients experience intermittent pain, making the diagnosis even more difficult, being attributable to episodes of torsion and detorsion, separated by asymptomatic intervals^(1,8)^.

The differential diagnosis of ovarian torsion includes appendicitis, diverticulitis, renal colic, pelvic inflammatory disease, corpus luteum cyst, and endometriosis^(2,9-14)^. The definitive diagnosis is made on the basis of laparoscopic findings^(8)^.

## MRI FINDINGS

**Figure 1:**
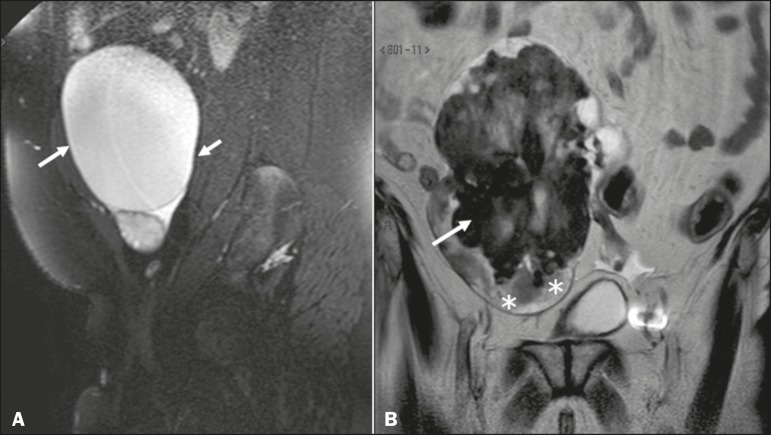
Sagittal and coronal T2-weighted MRI sequences (**A** and **B**, respectively). **A:** Preserved ovarian volume and an adjacent hyperintense expansile cystic formation consistent with a paraovarian cyst (arrows). **B:** Image of another patient with an enlarged ovary due to expansile, hypointense, heterogeneous fibroma-like formation (arrow). Note also the edema at the periphery of the ovarian parenchyma (asterisks).

**Figure 2:**
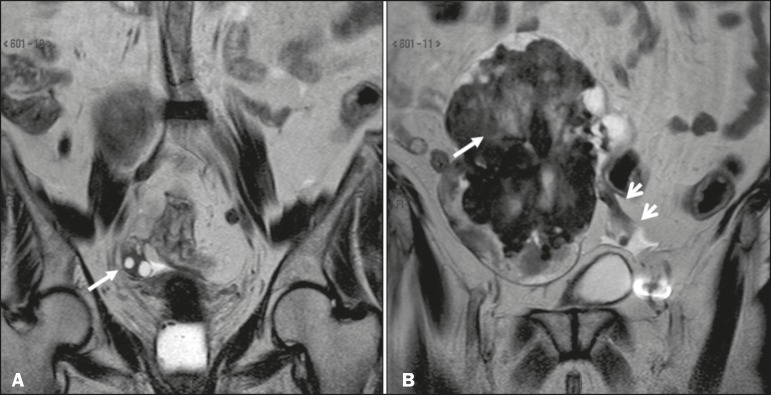
Coronal T2-weighted MRI sequences. **A:** Right ovary in a normal position with normal volume (arrow). **B:** Enlarged left ovary shifted to the right due to the presence of hypointense, heterogeneous expansile formation consistent with a fibroma (long arrow). Note also the ectatic vascular pedicle (short arrows).

**Figure 3:**
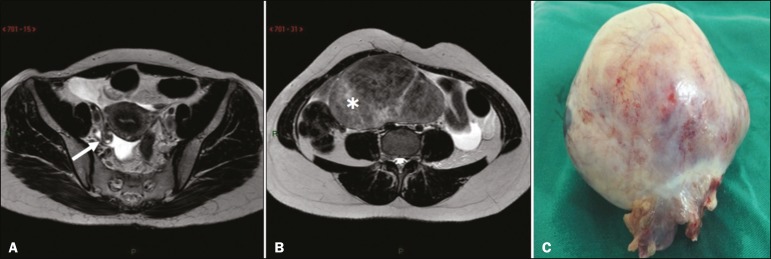
Axial T2-weighted MRI sequences. **A:** Right ovary in a normal position with normal volume (arrow). **B:** Enlarged left ovary shifted to the right due to the presence of hypointense, heterogeneous expansile formation consistent with a fibroma or fibrothecoma (asterisk). **C:** Surgical specimen showing a lobulated lesion that proved to be a fibrothecoma.

The main predisposing factor for ovarian torsion is the presence of a solid or cystic adnexal lesion, observed in up to 60% of cases, especially if the lesion is larger than 5.0 cm, functional cysts and mature cystic teratoma being the most common such lesions^(1,15-18)^. There have also been reports of cases of ovarian torsion predisposed by paraovarian cysts (Figure 1A), by ovarian cysts in the context of ovarian hyperstimulation syndrome^(17)^, and by corpus luteum cysts during the first trimester of pregnancy^(4)^. Among the solid adnexal lesions, fibrotic masses are notable for their heterogeneous aspect and markedly hypointense signal in T2-weighted MRI sequences (Figure 1B). In the context of ovarian torsion, such lesions typically shift toward the midline (in the contralateral direction), as shown in Figures 2 and 3.

**Figure 4:**
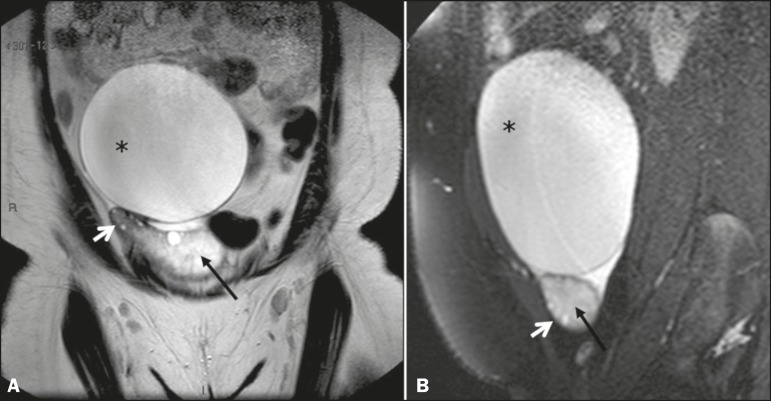
Coronal and sagittal T2- weighted MRI sequences (**A** and **B**, respectively). **A:** Large adnexal cystic formation (paraovarian cyst, asterisk). Note the hyperintense area, consistent with stromal edema, in the center of the ovary (black arrow), with consequent displacement of follicles to the periphery (white arrow). **B:** Another large adnexal cystic formation (paraovarian cyst, asterisk) . Again, there is a hyperintense area, consistent with stromal edema, in the center of the ovary (black arrow), with consequent displacement of follicles to the periphery (white arrow).

The most common radiological finding in ovarian torsion, albeit nonspecific, is that of an enlarged ovary (> 4.0 cm at its largest diameter), with signs of stromal edema or hemorrhage, characterized by high signal intensity in T2-weighted sequences in its central portion, with peripheral distribution of its follicles^(19)^, as depicted in Figure 4.

**Figure 5:**
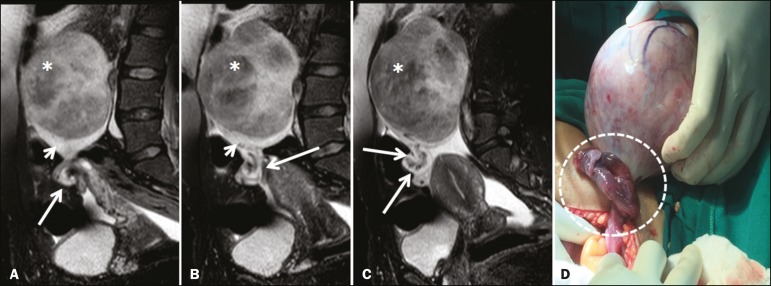
**A–C:** Sagittal fat-suppressed T2-weighted SPIR MRI sequences. Note the voluminous mass within the left ovary (asterisks), resulting in adnexal torsion, characterized by the whirlpool sign, with associated flow voids (long arrows), indicative of vascular torsion. The affected ovary (asterisk) is elevated and shows peripheral edema (short arrows). **D:** Intraoperative image showing the twisted, slightly ischemic adnexal pedicle (dashed outline) and the ovarian lesion that was later identified as a fibrothecoma.

**Figure 6:**
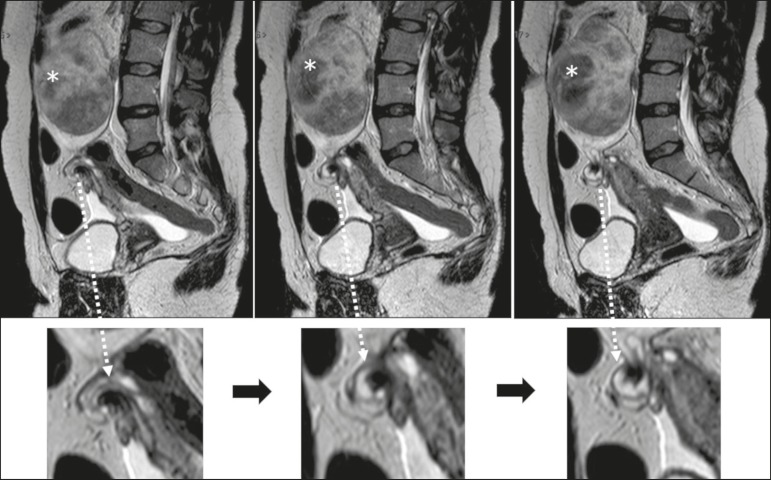
Sagittal T2-weighted MRI sequences, with magnified images below. Note that the ovarian pedicle is twisted around its own axis. The left ovary is enlarged due to the presence of a hypointense, heterogeneous expansile formation consistent with a fibroma (asterisks).

**Figure 7:**
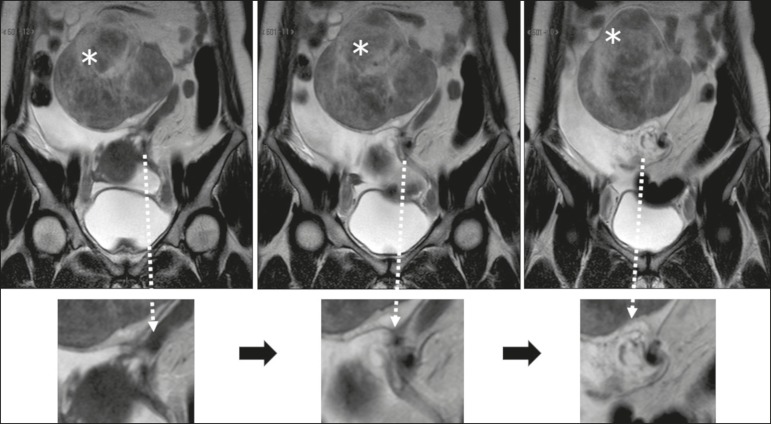
Coronal T2-weighted MRI sequences, with magnified images below. Note that the ovarian pedicle is twisted around its own axis. The left ovary is enlarged due to the presence of a hypointense, heterogeneous expansile formation consistent with a fibroma (asterisks).

The sign considered pathognomonic for ovarian torsion, albeit often difficult to recognize, is rotation of the ovarian vascular pedicle-the “whirlpool sign”-which is identified in less than one third of patients undergoing computed tomography or MRI^(20,21)^. Contrast-enhanced images obtained in the coronal and sagittal planes facilitate detection of the whirlpool sign^(8)^, as well as being useful for identifying flow voids within the adnexal structure, in order to trace the path of the blood vessels (Figure 5). As can be seen in Figures 6 and 7, a twisted vascular pedicle can be better characterized on T2-weighted MRI sequences of the flow voids, acquired in different planes.

**Figure 8:**
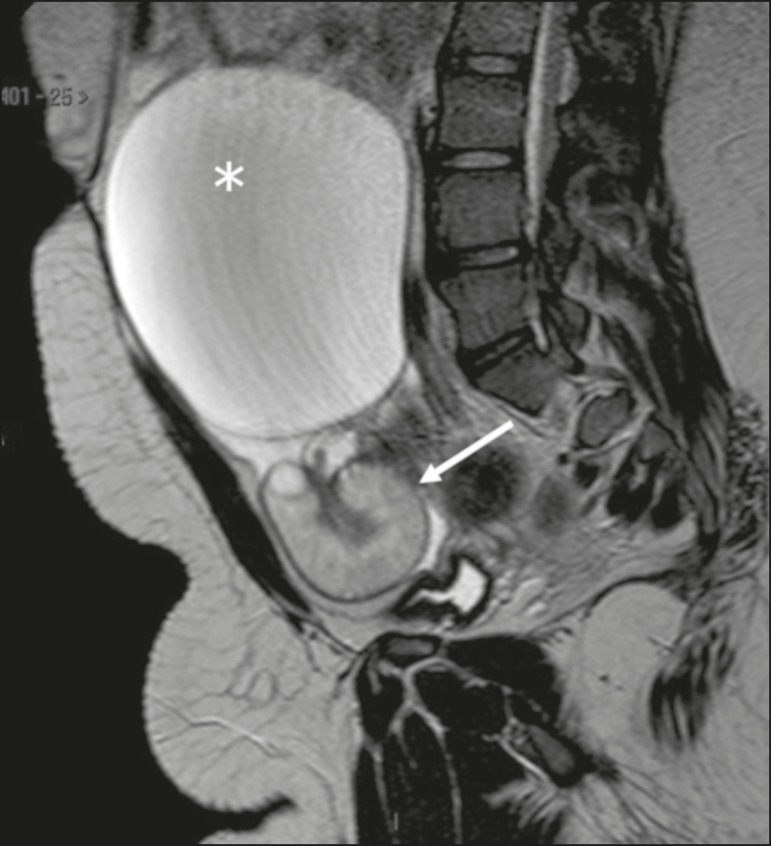
Sagittal T2-weighted MRI sequence. Thickened fallopian tube, measuring 1.4 cm in diameter (arrow). Note the large adnexal cystic formation (paraovarian cyst, asterisk).

Thickening of the uterine horn is a common, relevant feature for the diagnosis of ovarian torsion, characterized on imaging by a heterogeneous mass with intermediate signal intensity in T2-weighted sequences, sometimes assuming a “bull’s-eye” appearance, in close contact with an adnexal mass (Figure 8). In a case series including 25 patients, the prevalence of uterine horn thickening in the setting of ovarian torsion was found to be 84%, the most common presentation being a thickened tubular structure (in 60%), followed by the bull’s-eye aspect (in 8%), the latter typically being located between the adnexal mass and the uterus^(18)^.

Another feature that, when present, strongly supports a diagnosis of ovarian torsion is the detection of ovarian hemorrhage, in the form of an ovarian hematoma, hematosalpinx, or hemoperitoneum. The extent of the hemorrhage depends on the degree and duration of torsion, hemorrhagic infarction occurring at a later stage. In T1-weighted MRI sequences, a hyperintense rim within the ovary, although not unique to ovarian hemorrhage, should raise the suspicion of evolution to hemorrhagic infarction, depending on the clinical context^(8)^. Similarly, the pattern of enhancement of the ovarian parenchyma should be carefully studied. Persistent homogeneous enhancement-attributable to intermittent or very recent torsion-does not exclude a diagnosis of ovarian torsion, although minimal heterogeneous enhancement or even a lack of enhancement should raise the suspicion of incipient ischemia^(8)^.

## CONCLUSION

Albeit challenging, there is a need to distinguish between ovarian torsion and other diagnoses in patients with an adnexal lesion who develop acute pelvic pain. Therefore, radiologists should be familiar with the anatomy of the pelvis in its normal state and in the presence of adnexal lesions.
